# Effectiveness of renal denervation in the treatment of hypertension: a literature review

**DOI:** 10.1186/s40885-022-00194-6

**Published:** 2022-04-15

**Authors:** Riya Tejas Shah, Brian Xiangzhi Wang

**Affiliations:** 1 Department of Medicine, King’s College London, London, UK; 2 Department of Metabolism, Digestion and Reproduction, Faculty of Medicine, Imperial College London, London, UK

**Keywords:** Cardiovascular diseases, Clinical trial, Hypertension, Denervation, Ablation technique

## Abstract

**Background:**

Catheter-based renal denervation has been studied as a potential therapeutic option to reduce high blood pressure (BP). Preclinical studies in some experimental models have demonstrated an antihypertensive effect of renal denervation but reports from clinical trials have been mixed

**Methods:**

We performed a literature search using combinations of the key terms ‘Cardiovascular diseases, Clinical trial, Pre-clinical trials, Resistant hypertension, Renal denervation, Ablation technique, Radiofrequency ablation, Ultrasound ablation, RADIANCE SOLO, SYMPLICITY HTN, SYPRAL HTN’. The databases searched were PubMed and OVID Medline.

**Results:**

The initial SYMPLICITY HTN-1 AND HTN-2 clinical trials reported significant decreases in office BP but results from the more robustly designed SYMPLICITY HTN-3 trial, which included sham controls and ambulatory BP monitoring, showed no significant antihypertensive effect. Interest in the use of renal denervation in hypertension was once again sparked by favourable results from the SPYRAL HTN-OFF Med trial

**Conclusion:**

We provide a thorough, critical analysis of key preclinical and clinical studies investigating the efficacy of catheter-based renal denervation as a treatment for hypertension and highlight future areas for research to allow better translation into clinical practice

## Background

Hypertension is a global epidemic affecting 1.13 billion adults and is the most important risk factor of cardiovascular disease [1]. In the UK, hypertension is defined as blood pressure (BP) ≥140/90 mmHg [2]. Despite effective pharmacological therapy, only 20 to 80% of hypertensive patients have controlled BP. Hence, research into the prevention and treatment of hypertension is essential to reduce the global burden of disease.

Antihypertensive medication, alongside lifestyle modification, is the mainstay treatment for hypertension and is associated with a reduction in the risk of cardiovascular events (CVEs). Treatment for hypertension is patient-tailored, generally consisting of three main antihypertensive drug classes: angiotensin-converting enzyme inhibitors/angiotensin II receptor blockers, calcium-channel blockers, and diuretics [2, 3].

Approximately 10% of patients have resistant hypertension (BP ≥ 140/90 mmHg) despite using a diuretic and ≥ 2 additional antihypertensive drugs [4, 5]. Moreover, medication adherence is often poor (< 50% after 1 year of treatment) [6, 7]. Hence, a significant number of patients remain at risk of adverse CVEs, justifying the development of novel therapies.

**Fig. 1:**
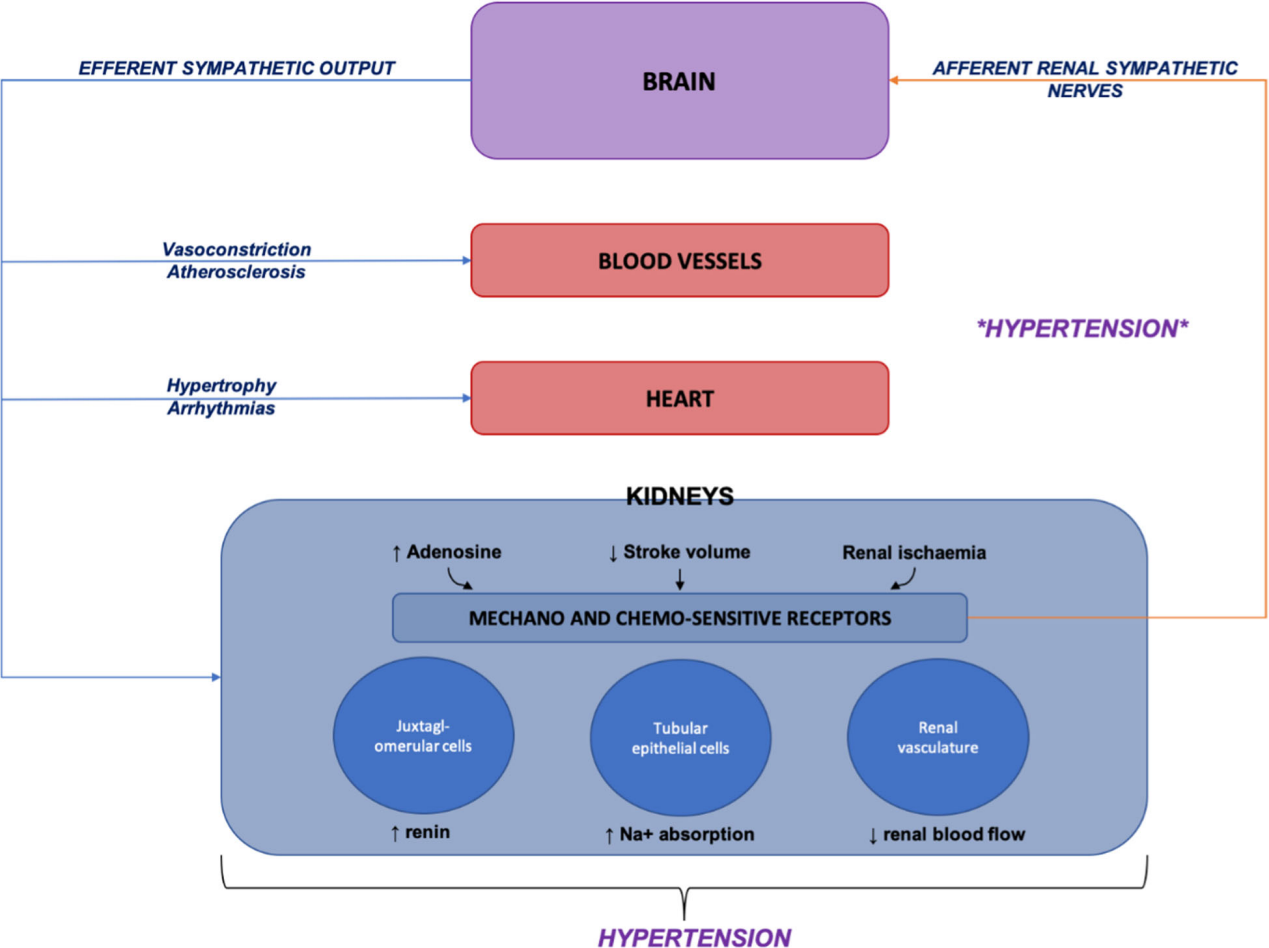
The role of renal sympathetic nerves in the pathology of hypertension

**Fig. 2:**
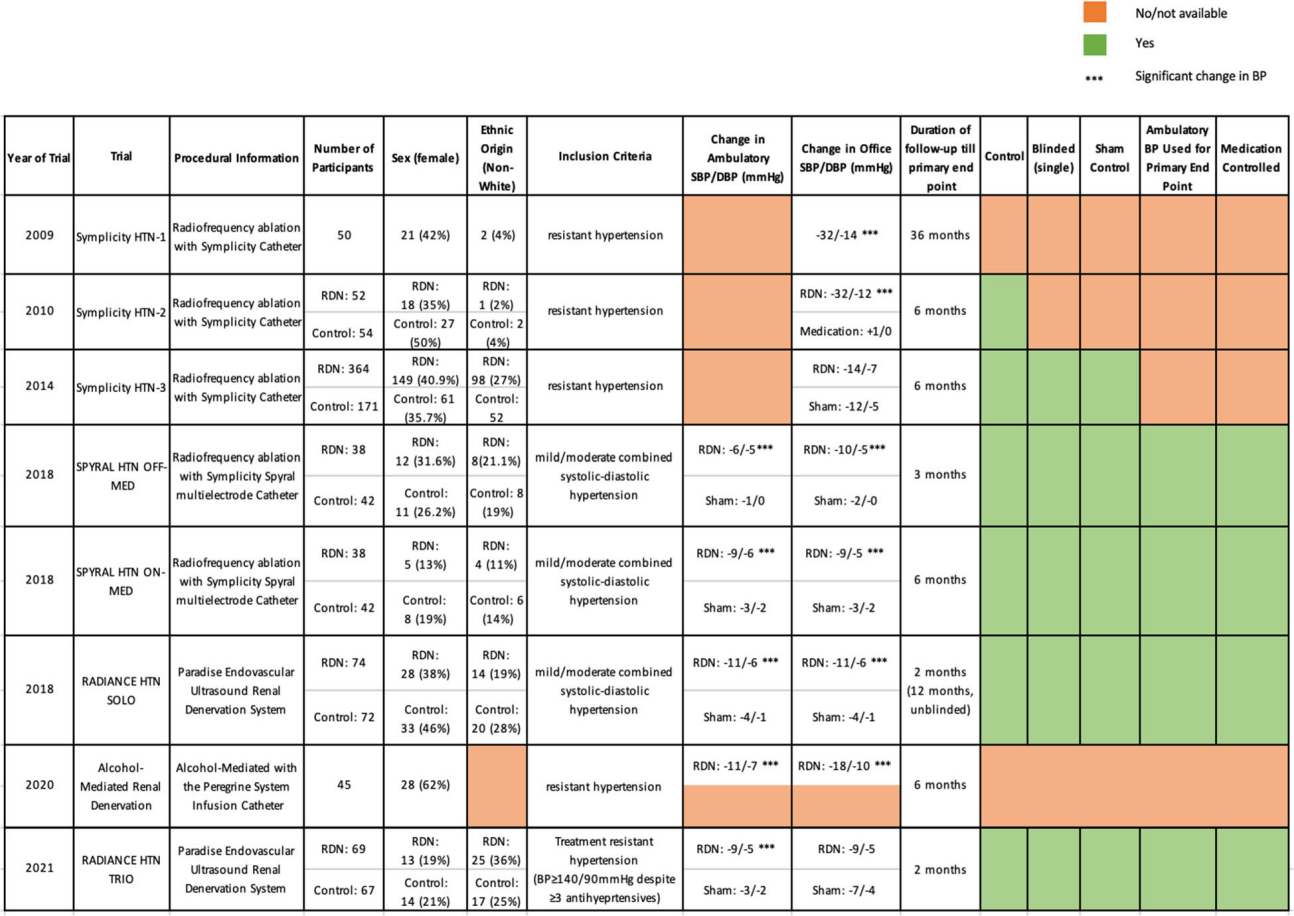
A summary of clinical trials mentioned in this paper and a visual representation of improvements in trial design. BP, blood pressure; SBP:, systolic blood pressure; DBP, diastolic blood pressure; RDN, renal denervation

Increased sympathetic activity in the afferent and efferent pathways between the kidneys and central nervous system contributes to the development of essential hypertension (Fig. 1). Increased understanding of the role of renal sympathetic nerves in the pathophysiology of hypertension (Fig. 2), led to the development of catheter-based renal denervation (RDN) via radiofrequency (RF), ultrasound energy, and alcohol [8]. These interventional therapies aim to disrupt the renal sympathetic pathways that contribute to hypertension.

We performed a literature search using combinations of the key terms ‘Cardiovascular diseases, Clinical trial, Pre-clinical trials, Resistant hypertension, Renal denervation, Ablation technique, Radiofrequency ablation, Ultrasound ablation, RADIANCE SOLO, SYMPLICITY HTN, SYPRAL HTN’. The databases searched were PubMed and OVID Medline. The effectiveness of RDN as a treatment for hypertension and avenues for further investigation will be explored herein through a review of preclinical and clinical trials.

## Main text

### Renal denervation

#### The rationale behind renal denervation evidence from preclinical trials

Bilateral surgical RDN in various experimental models of hypertension have demonstrated antihypertensive effects [9–11]. Studies in spontaneously hypertensive rats (SHR) report the most consistent findings with delayed onset or attenuation of hypertension [12–14]. The SHR is an established model of essential hypertension where increased renal sympathetic activity plays a pathogenic role, explaining the effectiveness of RDN in this model [10]. Similarly, bilateral surgical RDN in canines with sympathetically-mediated obesity can abolish hypertension and decrease plasma renin activity [15]. However, these findings are not always consistent and RDN does not always produce antihypertensive effects in certain experimental models including angiotensin II salt hypertensive rats, Wistar rats, and canines with hypertension induced by chronic nitric oxide synthase inhibition [16–18]. This suggests that RDN may be effective in certain forms of hypertension with scope for more personalised disease management in patients [12].

RDN via catheter-based RF ablation (RDN through bilateral RF ablation) (RF-RDN) allows for minimally invasive and targeted RDN, appropriate for translation into clinical practice. Hence, recent preclinical studies have investigated the efficacy and mechanisms of action of RF-RDN in more detail.

Gao et al. [19] conducted a robust sham-controlled trial in SHR testing the effects of bilateral RF-RDN procedurally similar to that done in humans. The RF-RDN group showed a sustained decrease in BP, measured using radio-telemetry, with a significantly lower systolic BP and diastolic BP 8-week post-ablation compared to sham controls. The observed BP reduction was not significantly different from that of SHR undergoing total denervation (surgical denervation + RF-RDN) suggesting that RF-RDN can achieve a maximal BP reduction. However, deeper analysis revealed greater variation in BP reduction between individual rats in the RF-RDN group compared to the total denervation group [19]. This hints at the impact of procedural variability on effectiveness of RF-RDN, important for translation into clinical practice.

As confirmation of decreased sympathetic activity, spectral density analysis showed a decrease in the low-frequency sympathetic component of BP variability in the RF-RDN group at 1- and 2-months post-ablation compared to sham controls. This was accompanied by an attenuated hypotensive response to a ganglionic blocker and a marked and sustained decrease in right and left kidney cortical norepinephrine levels in the RF-RDN group [19]. The markedly decreased norepinephrine levels also confirmed successful denervation. This conclusively links the observed BP reduction to a decrease in sympathetic activity and supports the efficacy of RF-RDN as a treatment for essential hypertension.

### Landmark clinical trials

#### SYMPLICITY-HTN trials

Investigation into the clinical viability of RDN in the treatment of resistant hypertension was spurred by the promising results from the initial proof-of-principle SYMPLICITY HTN-1 and randomised controlled HTN-2 clinical trials.

SYMPLICITY HTN-1 was a multi-centre, open-label cohort study that reported significant progressive and long-term reductions in SBP and DBP after RF-RDN (BP reduction: − 32.0/ -14.4 mmHg 36 months post-ablation) [20]. However, limitations included lack of blinding and a control group to confidently attribute the observed effects to RF-RDN. Moreover, patients remained on prescribed antihypertensive medication, and without a control group, the contribution of a possible Hawthorne effect could not be eliminated. Protocol understandably allowed for changes to prescribed antihypertensive medication in the extended follow-up period but this may have resulted in a more effective regime and a resultant BP reduction. Furthermore, office BP, used in this study, is not reliable and is susceptible to the phenomenon of white coat hypertension. Over the extended follow-up period, patients may have become acclimatised to office BP measurements resulting in a diminished white coat effect and a reduction in BP undistinguishable from that caused by the procedure. Moreover, only 5% of the study population was of Non-White ethnic origin thus the results were not generalisable to individuals of other ethnicities.

In terms of safety, no major procedure-related clinical complications were reported and there were no episodes of vasovagal syncope or orthostatic hypotension in contrast to the fall in mean arterial pressure (MAP) reported in an ovine study of RDN by Singh et al. [21]. However, renal function declined in 28 patients who experienced a fall in estimated glomerular filtration rate (GFR) > 25% at least once after RDN. Importantly, 4 patients were investigated for renal artery stenosis with one new case requiring stenting. Post-denervation renal artery stenosis has been reported previously and poses a possible safety risk leading to renal impairment and increased cardiovascular risk.

Finally, durability of BP reduction was demonstrated by a persistent reduction from the first follow-up at 1 month to the extended follow-up at 36 months (SBP 1 month: − 18.9 mmHg, standard deviation [SD] 19.2; 36 months: − 32.0 mmHg, SD 7.6). However, there was a significant dropout rate with 141 patients at 1 month to 88 patients at 36 months possibly resulting in selection bias.

In SYMPLICITY HTN-2, the RDN group also demonstrated a long-term and significantly higher BP reduction than the control group. However, limitations in study design such as use of office BP to measure the primary endpoint, lack of blinding, and lack of a sham control meant that the results still did not robustly support the efficacy of RF-RDN in the treatment of hypertension.

The SYMPLICITY HTN-3 trial was designed to overcome some of these limitations and provide a more definitive assessment of RF-RDN. No significant difference in 6-month post-ablation mean office SBP reduction or 24-h ambulatory SBP was observed between RF-RDN and sham groups [22]. However, multivariate analysis identified aldosterone antagonist use, non-use of vasodilators, and a baseline office SBP ≥180 mmHg as positive predictors of office SBP reduction [23]. Moreover, there was no significant difference in 24-h ambulatory SBP reduction between African American and Non-African American participants. However, African American sham control participants had a significantly greater reduction in office SBP compared to Non-African American controls-possibly as a result of an exacerbated Hawthorne effect. This suggests that RDN may be more effective in selected patient populations but also stresses the importance of using ambulatory BP for reliable readings [23].

The SYMPLICITY HTN-3 trial included a larger cohort of 535 patients, greater racial diversity, ambulatory BP, and, most importantly, a sham control. Despite these improvements in design, patients had a high mean pill burden and medical regimes were altered in 39% of patients up till the primary end point [23, 24]. These confounding factors could have made it harder to observe significant BP reductions.

Another critical failure of the study was ineffective RDN. Eighty-eight centres were recruited for the trial, increasing the likelihood of inter-operator variability. Moreover, HTN-3 trial operators were inexperienced and analysis revealed that only 19 treated patients received recommended circumferential ablation [23, 25]. Hence, the lack of significant BP reduction in the early SYMPLICITY trials did not necessarily disprove RDN’s efficacy.

#### SPYRAL HTN-OFF MED and ON MED trials

The recent multinational SPYRAL HTN trials sought to develop further from the SYMPLICITY trials. The OFF-MED trial assessed the efficacy of RDN in hypertensive patients who were not on pharmacological medication and the ON-MED trial assessed the efficacy of combined pharmacological and RDN treatment in hypertensive patients [26].

Both trials demonstrated significantly greater 24-h ambulatory BP changes from baseline in the RDN group compared to sham controls. The mean difference in BP change between the two groups was − 5.00 mmHg SBP, − 4.4 mmHg DBP in the OFF-MED trial and − 7.4 mmHg SBP, − 4.1 mmHg DBP in the ON-MED trial [27, 28]. Though significant, these reductions in BP were considerably less than the initial SYMPLICITY trials. Further long-term results of the ON-MED trial with a larger cohort are awaited [29].

Both the SPYRAL HTN-OFF MED and ON MED trials consisted of sham controls and used a multielectrode catheter that automatically enables 4-quadrant RF ablation for circumferential treatment. Additionally, each centre had a maximum of 1 operator supervised by experienced instructors who encouraged adherence to recommended ablation protocols. Combined, this aimed to reduce the variability and increase the effectiveness of RDN.

The confounding effects of medication was eliminated in the OFF-MED trial and reduced in the ON-MED proof-of-concept trial by limiting pharmacological treatment to a maximum of 3 standardised medications. Adherence to these medication regimes was assessed in both trials by urine and blood analysis but was only 60% in the ON-MED proof-of-concept trial, which means that effectiveness of RDN cannot be ascertained in the medication adherent population [28]. Interestingly, a post-hoc analysis of the ON-MED trial reported a statistically significant reduction in the rate of morning DBP surge, but not SBP surge [30]. The rate of the morning surge in BP is a risk factor for CVE such as myocardial infarction and stroke thus this attenuation in the DBP morning surge may be clinically important [31].

Unlike previous clinical trials, the SPYRAL trials only included patients with moderate combined systolic-diastolic hypertension and excluded patients with more severe and isolated systolic hypertension in whom comorbidities and high medication burden made it difficult to reliably assess treatment effectiveness. However, exclusion of these groups means that the results cannot be generalised. With a short duration of 3 and 6 months, longer term follow-up is essential to establish durability of antihypertensive effects.

#### RADIANCE-HTN SOLO

Much like the SPYRAL trials, the RADIANCE-HTN SOLO trial was an international, sham-controlled, single-blinded randomised study that assessed RDN’s effectiveness using the Paradise catheter system (ReCor Medical) in patients with combined moderate systolic-diastolic hypertension. Patients were taken off medication 4 weeks before randomisation and remained off-medication for 2 months post-procedure. There was a significantly greater reduction in daytime ambulatory SBP at 2 months post-procedure in the RDN group compared to the sham group. This positive response to RDN was seen across sex, ethnicity, geography, and varying baseline BP [32]. Again, these reductions were smaller than anticipated from the SYMPLICITY trials but are still clinically significant.

The major difference in the RADIANCE-HTN SOLO trial was that renal nerve ablation was achieved using ultrasound energy, which may be superior to renal denervation through bilateral RF ablation [33];. As in the SPYRAL OFF-MED trial, confounding effects of medication were removed by eliminating medications. However, adherence was only monitored through patient and physician reporting rather than objective assessment with urine and blood analysis. Moreover, 55% of treated patients had to return to medication after 2 months due to insufficient BP control, questioning the durability of ultrasound RDN as a stand-alone treatment [32]. Interestingly, an author of the study presented the findings from an analysis of 31 sham patients who crossed over to receive RDN. In this group, there was a 12.2 mmHg reduction in daytime ambulatory SBP 6 months post-denervation. However, participants and physicians were unblinded at crossover possibly introducing confounding effects as seen in the early SYMPLICITY trials [34]. The 12-month results of the RADIANCE-HTN SOLO trial demonstrated a maintained reduction in daily ambulatory SBP. There was no significant difference in daily ambulatory SBP between RDN and sham-control groups at this time-point, but the RDN group did have a significantly lower number of prescribed antihypertensive medications. This highlights the potential for RDN to reduce medication burden which has implications in terms of medication adherence and health maintenance to prevent disease progression [35].

Two trials assessing the efficacy of ultrasound-based RDN have been planned including the REQUIRE trial, with a cohort of drug-resistant hypertensive patients, and the RADIANCE II trial, with a cohort of patients with uncontrolled hypertension [36].

#### Alcohol mediated renal denervation

Recently, a small, multicentre, open-label clinical trial assessing the efficacy of an alcohol-mediated RDN system demonstrated a − 11 mmHg reduction in mean 24-h ambulatory SBP 6 months post-denervation. As with the early SYMPLICITY trials, lack of blinding, a control group, and also a small study cohort mean that the results cannot be reliably confirmed. A more thorough study design is needed to assess the efficacy and safety of alcohol-mediated RDN. If proven to be effective, potential benefits of alcohol-mediated RDN may include increased simplicity of setup and a reduction in cost of the therapy [37].

#### Clinical trials and the European Society of Hypertension position on renal denervation

Early clinical trials did not consistently and reliably demonstrate a reduction in BP with RDN. However, recent trials have taken lessons from these early trials to create more robust methodologies and incorporate a more representative study population. Multiple systematic reviews have concluded these recent studies demonstrate a modest but clinically meaningful reduction in BP [38, 39]. As such, the 2021 European Society of Hypertension (ESH) position paper on RDN takes a stance in support of RDN. The position paper affirms the safety of RDN and its use as a potential adjunctive therapeutic option in the treatment of hypertension. Moreover, it suggests further investigation into patients’ and physicians’ perspectives of RDN to gauge potential uptake and treatment preferences which would help guide the development of a treatment pathway which incorporates RDN [40]. However, this literature review has also demonstrated intra and inter trial variability in BP reduction which points to avenues for further research also highlighted in the ESH position paper. These include predictors of significant response to RDN therapy to identify patients who will benefit most from treatment, factors leading to improved procedural efficacy, efficacy of RDN in the presence of comorbidities, and a more direct comparison of different RDN techniques. Though these questions are still to be investigated in clinical trials, preclinical trials may provide some insight.

### Insight from preclinical studies into gaps in knowledge

#### Response with co-morbidities

##### Obesity

Obesity affects an estimated 28% of adults in England and is strongly linked to hypertension in humans [41, 42]. Studies have implicated increased renal sympathetic activity in the pathogenesis of obesity-induced hypertension, indicating that RF-RDN may be valuable for these patients [41, 43].

The obese hypertensive dog is a large animal with systemic and metabolic changes similar to those seen in obese humans thus is a valuable model to test RF-RDN in obesity-induced hypertension [44]. A study testing RF-RDN with the St. Jude Medical EnligHTN system (St. Jude Medical, Saint Paul, MN, USA) in obese hypertensive dogs showed significant, sustained reductions in MAP and SBP ≥10 mmHg in all dogs. The procedure achieved only partial denervation with injury to 46% of observed renal nerves 8 weeks after ablation and a 6-week post-ablation renal norepinephrine level 42% less than that in normotensive dogs. This suggests that antihypertensive effects can be achieved even with partial denervation.

Moreover, no change in GFR was observed suggesting the procedure has no detrimental impact on renal function [44], a finding supported in other experimental models [21, 45].

##### Hypertensive chronic kidney disease

Another study by Singh and colleagues demonstrated the effectiveness of RF-RDN in an ovine model of hypertensive chronic kidney disease. A reduction in BP to normotensive levels and a greater improvement in GFR and renal blood flow 5 months post-ablation was observed in hypertensive CKD RF-RDN groups compared to sham controls. However, in response to haemorrhage, there was a greater fall in MAP in the RF-RDN groups [21]. This suggests that compensatory mechanisms in response to haemodynamic challenges may be compromised after RDN. This has important clinical implications with potentially more risk during surgery, trauma, and gastrointestinal bleeding.

#### Determinants of procedural efficacy

Differences in RF-RDN procedural technique is another aspect that may explain the variances in BP reduction documented in preclinical and clinical trials [19, 46].

In a porcine model, combined targeted RF-RDN of both the distal main artery and branches resulted in the greatest and most consistent reduction in renal norepinephrine and cortical axon density indicating more complete denervation [47]. This is explained by examination of renal nerve distribution where renal nerves in the distal artery are closer to the arterial lumen than renal nerves in the proximal artery [48]. This suggests that targeted circumferential ablation would produce a more pronounced antihypertensive response and accentuates the importance of standardising RF-RDN techniques in clinical trials and treatment.

Alternatively, targeted ablation at sites where renal nerve stimulation elicits a clear rise in BP allows for a more individualised procedural approach. This approach was used in a canine model in which targeted ablation resulted in a significant reduction in BP and plasma norepinephrine 3 months post-denervation [49].

#### Durability

Anatomical and functional reinnervation has been reported in numerous normotensive and hypertensive animals weeks to months after RDN [10, 50, 51]. In a study using the Symplicity Flex catheter (Medtronic, Inc., Santa Rosa, CA, USA) in normotensive sheep, the BP rise on electric stimulation of afferent and efferent renal nerves returned to predenervation levels after 11 months [52]. Supporting this, histopathologic analysis of 49 renal arteries in normotensive pigs post-RDN showed a gradual decline in acute nerve injury after 7 days and a gradual increase in focal nerve regeneration from 17% of renal arteries after 60 days to 71% after 180 days [53]. These studies point at the possibility of post-RDN reinnervation in humans and the presence of alternate mechanisms that sustain the BP reduction seen in clinical trials.

Preclinical studies allow for in depth anatomical and functional assessment providing a deeper insight into the science behind RDN. However, animals in preclinical studies are relatively homogenous and may not account for confounding factors present in humans. Moreover, hypertension already has a good treatment base in pharmacological therapy and the therapeutic value of RDN can only be discerned when tested against or as an adjunct to medication.

### Future directions

Preclinical studies show robust evidence of heightened sympathetic activity in hypertension and subsequent reduction of this activity by catheter-based RDN across a wide range of species and experimental models. This provides theoretical backing for RDN which favours its effectiveness in the treatment of hypertension. However, failure to replicate antihypertensive effects in certain animal models points at a possible need to identify patients most suited to RDN. In particular, RDN had a substantial antihypertensive effect in experimental animal hypertension models with high sympathetic activity thus hypertensive patients with greater sympathetic dependence may prove ideal candidates [12, 54]. On the other hand, analysis focusing on patients from the Symplicity HTN-3 trial with isolated systolic hypertension showed a lower efficacy compared to patients with combined systolic/diastolic hypertension [55]. This further emphasises the importance of further research to ensure the procedure is targeted towards those who will benefit the most from it.

Results from the recent SPYRAL-HTN OFF MED and RADIANCE-HTN SOLO trials have resurrected enthusiasm for catheter-based RDN. However, longer term follow-up and awaited results from the SPYRAL-HTN ON MED trial are still required to ensure durability of treatment and efficacy with antihypertensive medication. Nevertheless, the potential of catheter-based RDN as a stand-alone treatment foregoing the need for constant intake of antihypertensive medication and its side effects is in itself appealing.

Another common theme seen in clinical trials is the variation in BP response which is possibly linked to differences in procedural success. These variances have highlighted the need to develop methods to monitor and ensure effective RDN for future trials and, potentially, clinical practice. Another potential method to ensure successful denervation is eliciting renal vasoconstriction via a reflex mechanism in response to a stimulus such as isometric handgrip. The stimulus excites efferent renal sympathetic nerves and thus this method can be used to measure and compare renal sympathetic activity before and after the intervention. Finally, developing technologies can also be used to passively measure renal sympathetic nerve traffic and confirm successful denervation [54]. These methods can potentially be incorporated into future trials to confirm successful denervation and, if BP variability still persists, assess its causative factor.

## Conclusions

**Fig. 3:**
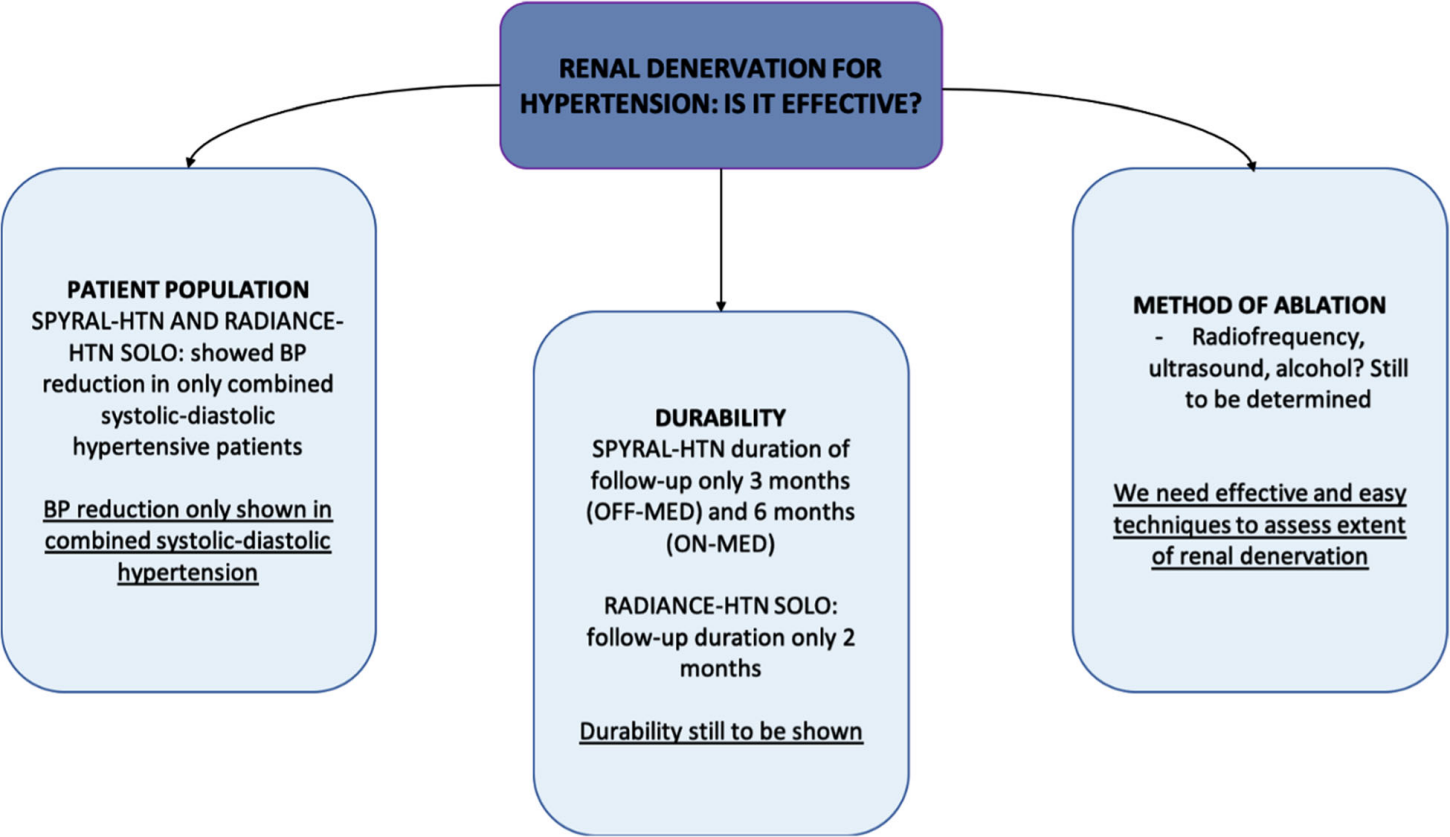
Current position on the effectiveness of renal denervation highlighting three factors for further study. BP, blood pressure

With a scientific basis evidenced by preclinical trials and positive outcome in recent clinical trials, there is substantial support for continued investigation of RDN in the treatment of hypertension. However, durability, method of ablation, and target population need to be further investigated before RDN can be used in clinical practice (Fig. 3). If successful, RDN can have a profound impact on cardiovascular and global health.

## Data Availability

Not applicable.

## Abbreviations

BP: Blood pressure
CVE: Cardiovascular event
ESH: European Society of Hypertension
GFR: Glomerular filtration rate
MAP: Mean arterial pressure
RDN: Renal denervation
RF: Radiofrequency
SD: Standard deviation
SHR: Spontaneously hypertensive rats
